# RETRACTION: Assessing Biomarkers for Post‐Surgical Wound Healing: A Meta‐Analysis of Exosome‐Based CircRNA in Breast Cancer Recovery

**DOI:** 10.1111/iwj.70534

**Published:** 2025-04-18

## Abstract

**RETRACTION:** ZhangY. , TaoK. , DingL. , and ZhaoY. , “Assessing Biomarkers for Post‐Surgical Wound Healing: A Meta‐Analysis of Exosome‐Based CircRNA in Breast Cancer Recovery,” International Wound Journal 21, no. 2 (2024): e14723, 10.1111/iwj.14723.38379248 PMC10830351

The above article, published online on 31 January 2024, in Wiley Online Library (http://onlinelibrary.wiley.com/), has been retracted by agreement between the journal Editor in Chief, Professor Keith Harding; and John Wiley & Sons Ltd. Following an investigation by the publisher, all parties have concluded that this article was accepted solely on the basis of a compromised peer review process. The editors have therefore decided to retract the article. The authors did not respond to our notice regarding the retraction.

**RETRACTION:** Y. Zhang , K. Tao , L. Ding , and Y. Zhao , “Assessing Biomarkers for Post‐Surgical Wound Healing: A Meta‐Analysis of Exosome‐Based CircRNA in Breast Cancer Recovery,” International Wound Journal 21, no. 2 (2024): e14723, 10.1111/iwj.14723.38379248 PMC10830351

The above article, published online on 31 January 2024, in Wiley Online Library (http://onlinelibrary.wiley.com/), has been retracted by agreement between the journal Editor in Chief, Professor Keith Harding; and John Wiley & Sons Ltd. Following an investigation by the publisher, all parties have concluded that this article was accepted solely on the basis of a compromised peer review process. The editors have therefore decided to retract the article. The authors did not respond to our notice regarding the retraction.

